# Other primary malignancies are associated with inferior survival in multiple myeloma: a single-center matched retrospective cohort study

**DOI:** 10.3389/fonc.2026.1943171

**Published:** 2026-09-11

**Authors:** Changnian Li, Yaru Wang, Lulu Li, Zezhou Zang, Zhaoxia Lin, Qingyuan Zhang, Ruirong Xu, Siyuan Cui

**Affiliations:** 1 First Clinical Medical College, Shandong University of Traditional Chinese Medicine, Jinan, China; 2 Department of Hematology, Affiliated Hospital of Shandong University of Traditional Chinese Medicine, Jinan, China

**Keywords:** matched cohort, multiple myeloma, other primary malignancy, overall survival, real-world study, treatment sequencing

## Abstract

**Background:**

Other primary malignancies (OPMs) may complicate diagnostic assessment, treatment sequencing, and prognostic evaluation in multiple myeloma (MM). This study characterized the spectrum and timing of OPMs and evaluated their association with overall survival (OS).

**Methods:**

We retrospectively screened 461 unique patients with MM treated or followed at a single center between August 8, 2019, and June 6, 2026. Patients with OPMs were categorized as OPM-first, synchronous, or MM-first and matched 1:2 with patients without OPMs by sex, age at MM diagnosis, and year of MM diagnosis. OS was evaluated using Kaplan–Meier analysis, matched-set-stratified log-rank testing, and stratified Cox regression.

**Results:**

Twenty-seven patients had OPMs, corresponding to a crude proportion of 5.9%. Lung cancer (37.0%) and prostate cancer (18.5%) were the most common OPMs. Ten patients (37.0%) were classified as OPM-first, 12 (44.4%) as synchronous, and 5 (18.5%) as MM-first. OPMs affected MM management in 29.6% of patients, whereas MM or anti-myeloma treatment affected OPM management in 18.5%; treatment was modified because of documented or anticipated overlapping toxicities in 29.6%. Median OS was 46.2 months in cases and was not reached in matched controls. OPMs were associated with inferior OS in the stratified Cox analysis (hazard ratio, 3.92; 95% CI, 1.35–11.37; *P* = 0.012), and the association persisted after MM-first cases were excluded.

**Conclusion:**

Lung and prostate cancers were the most common OPMs in this cohort, highlighting the need for heightened clinical attention to these malignancies in patients with MM. The presence of an OPM was associated with inferior OS; however, this finding should be interpreted cautiously because of the small sample size and marked tumor heterogeneity. When both malignancies require active treatment, multidisciplinary assessment is important to determine treatment priority according to disease activity, treatment urgency, and the patient’s overall condition.

## 1 Introduction

Multiple myeloma (MM) is a plasma cell malignancy that commonly presents with anemia, renal impairment, hypercalcemia, osteolytic bone disease, and recurrent infections (1). The introduction of proteasome inhibitors, immunomodulatory drugs, anti-CD38 monoclonal antibodies, autologous stem cell transplantation, and more recently T-cell–redirecting therapies has expanded treatment options and improved outcomes for patients with MM (2–4). As survival improves, second primary malignancies and other non-MM causes of morbidity and mortality are becoming increasingly relevant in long-term care (5).

Previous studies have mainly focused on malignancies arising after MM treatment, particularly the possible association between lenalidomide exposure and second primary malignancies (5–7). In clinical practice, however, another primary malignancy may be diagnosed before, concurrently with, or after MM. Distinguishing an independent primary tumor from metastatic disease, recurrence, or an extramedullary manifestation of MM can be difficult in some patients (8). Although multiple primary malignancies are commonly classified as synchronous or metachronous, the diagnostic interval used to define these categories varies among studies (9).

Single-center and population-based studies have described previous and subsequent malignancies in patients with MM, but data that integrate tumor spectrum, diagnostic timing, treatment sequencing, and survival relative to matched patients without an OPM remain limited (10–13). We therefore identified patients with MM and OPMs from a single-center cohort of 461 patients, described their clinical and temporal characteristics, and compared overall survival with that of matched patients with MM without an OPM.

## 2 Materials and methods

### 2.1 Study population

This was a single-center retrospective matched cohort study. The study population comprised patients with multiple myeloma (MM) who received inpatient care or follow-up at our center between August 8, 2019, and June 6, 2026. MM-related inpatient records were retrieved from the medical record system. After exact duplicate records were removed, records were deduplicated using unique patient identifiers to establish the MM screening cohort. For patients diagnosed with MM or another primary malignancy before the retrieval period, previous diagnostic, treatment, and follow-up information was collected retrospectively.

Within the screening cohort, cases were defined as patients with MM who also had another primary malignancy diagnosed before, concurrently with, or after MM. Each other primary malignancy (OPM) was required to have histopathologic confirmation and to be considered an independent primary tumor on the basis of medical records, imaging findings, and the overall clinical course, rather than an extramedullary manifestation of MM, a plasma cell–related lesion, recurrence of a previous malignancy, or metastasis from another malignancy.

To evaluate the association between OPMs and survival outcomes in patients with MM, matched controls were selected from contemporaneous patients with MM without an OPM, and a matched cohort was constructed.

### 2.2 Construction of the matched control group

Controls were selected from patients in the same screening cohort who had no OPM. To reduce the effects of age, sex, and diagnostic era on survival outcomes, each case was matched with 2 controls (1:2). Matching variables included sex, age at MM diagnosis, and year of MM diagnosis. Sex was required to be identical, age at MM diagnosis was matched within ±5 years, and year of MM diagnosis was matched within ±2 years.

Each control could be used only once and was not matched to more than 1 case. Control selection was not based on survival status, date of death, date of last follow-up, or duration of overall survival (OS). After matching, the cases and controls together constituted the matched cohort used for survival comparisons.

### 2.3 Eligibility criteria

The inclusion criteria were as follows: (1) a definitive diagnosis of MM documented in the medical records; (2) receipt of inpatient care or follow-up at our center during the study period; and (3) availability of the date of MM diagnosis, principal clinical data, and follow-up outcomes. Cases were additionally required to have an OPM diagnosed before, concurrently with, or after MM. The OPM had to be histopathologically confirmed and considered an independent primary malignancy on the basis of medical records, imaging findings, and the overall clinical course. Controls were required to have no documented diagnosis of an OPM in the available medical records and to have available matching variables and follow-up outcomes.

The exclusion criteria were as follows: (1) an imaging-detected mass, nodule, or suspicious lesion without confirmation of malignancy; (2) a lesion considered to represent an extramedullary manifestation of MM, extramedullary plasmacytoma, or another plasma cell–related disorder; (3) a lesion considered to represent metastasis from a previous or synchronous malignancy rather than an independent primary malignancy; (4) missing key diagnostic, treatment, or follow-up information that precluded the primary analyses; and (5) duplicate hospitalization or patient records.

### 2.4 Data collection

Data were collected from electronic medical records, hospital discharge records, laboratory information systems, pathology systems, imaging reports, and follow-up records. For both cases and controls, the following variables were collected: sex; age at MM diagnosis; date of MM diagnosis; M-protein type; light-chain type; hemoglobin level; bone marrow plasma cell percentage; International Staging System (ISS) stage; Revised International Staging System (R-ISS) stage; β2-microglobulin level; presence of extramedullary disease at MM diagnosis; frontline anti-myeloma regimen; use of proteasome inhibitor-, lenalidomide-, or anti-CD38 monoclonal antibody–containing regimens; receipt of autologous stem cell transplantation (ASCT); date of last follow-up; and survival status. Laboratory measurements obtained at the time of MM diagnosis or before treatment and closest to the date of diagnosis were preferentially used.

For cases, additional OPM-related information was collected, including the date of diagnosis, tumor site, histologic type, clinical or pathologic stage, presence of distant metastasis at diagnosis, OPM status at the time of MM diagnosis, principal treatment modality, receipt of surgery, receipt of systemic antineoplastic therapy, treatment sequencing between the two malignancies, and OPM status at the last follow-up. We also reviewed whether the OPM affected MM management, whether MM or anti-myeloma treatment affected OPM management, and whether treatment was delayed, reduced, interrupted, or otherwise modified because of documented or anticipated overlapping toxicities. Cause of death was recorded when available.

### 2.5 Definitions

According to the interval between the diagnoses of MM and the OPM, cases were classified into 3 groups. Patients in whom the OPM was diagnosed more than 2 months before MM were classified as OPM-first. Patients in whom the 2 malignancies were diagnosed within 2 months of each other were classified as synchronous. Patients in whom MM was diagnosed more than 2 months before the OPM were classified as MM-first. The diagnostic interval was calculated as the date of OPM diagnosis minus the date of MM diagnosis. Negative values indicated that the OPM preceded MM, whereas positive values indicated that MM preceded the OPM.

Extramedullary disease at MM diagnosis was recorded when contemporaneous medical records or imaging reports documented plasma cell involvement in soft tissue or an organ outside the bone marrow, excluding lesions attributed to the OPM or metastatic disease. OPM status at the time of MM diagnosis was classified as active, previously treated or controlled, or not yet diagnosed. Active OPM included newly diagnosed disease, ongoing treatment, residual disease, recurrence, progression, or metastatic disease at MM diagnosis. Previously treated or controlled OPM referred to a malignancy for which treatment had been completed and no active disease was documented at the time of MM diagnosis. OPMs diagnosed after MM were categorized as not yet diagnosed at MM diagnosis.

Distant metastasis was determined from the staging information, imaging reports, and medical records available at the time of OPM diagnosis. Patients with explicitly documented distant metastasis or other evidence of distant dissemination were classified as having distant metastasis. Local progression or regional lymph-node involvement without clear evidence of distant spread was not classified as distant metastasis. Diffuse large B-cell lymphoma was not included in the distant-metastasis classification used for solid tumors.

Treatment sequencing was determined according to the actual order in which treatment for the 2 malignancies was initiated and the principal treatment objective documented in the medical records. Treatment sequencing was categorized as MM-first treatment, OPM-first treatment, or concurrent treatment. Systemic antineoplastic therapy for the OPM was defined as chemotherapy, immunochemotherapy, targeted therapy, or other systemic anticancer treatment.

An OPM was considered to have affected MM management when its presence led to a documented change in treatment timing, regimen, treatment intensity, or the ASCT plan. Conversely, MM was considered to have affected OPM management when MM or anti-myeloma treatment led to postponement or modification of OPM surgery, or to delay, dose reduction, interruption, or modification of systemic treatment for the OPM. A treatment modification related to overlapping toxicity was recorded when treatment was changed because of a documented adverse event or an anticipated overlap in toxicity between the two treatment strategies. When the available records were insufficient to determine whether one malignancy affected the management of the other, the treatment influence was recorded as unknown.

### 2.6 Follow-up and outcomes

Follow-up data were obtained from outpatient records, inpatient records, follow-up documentation, and mortality records. The date of last follow-up was defined as the last date on which a definitive survival or disease-status assessment was available. For patients who died, the date of death was used as the event date. Patients who were alive at the last follow-up were censored on that date.

The primary outcome was OS in the matched cohort. For survival comparisons between cases and matched controls, OS was defined as the interval from the date of MM diagnosis to death from any cause or the date of last follow-up. Death was considered the event, and surviving patients were censored at the date of last follow-up.

### 2.7 Statistical analysis

Continuous variables were summarized as medians and ranges, and categorical variables as numbers and percentages. For descriptive comparisons of nonmatching baseline variables, continuous variables were compared using the Mann–Whitney U test, R-ISS stage using the Pearson χ² test, and binary categorical variables using Fisher’s exact test.

OS was estimated using the Kaplan–Meier method. Survival differences between cases and matched controls were assessed using both unstratified and matched-set-stratified log-rank tests. A Cox proportional hazards model stratified by matched set was used to estimate hazard ratios and corresponding 95% confidence intervals. Because of the limited number of deaths, multivariable Cox regression was not performed.

A sensitivity analysis was conducted after excluding MM-first cases and their corresponding matched controls. Among patients whose OPM had been diagnosed by the time of MM diagnosis, an exploratory comparison of OS between those with active OPM and those with previously treated or controlled OPM was performed using the Kaplan–Meier method and log-rank test. Lung cancer, the most common OPM subtype, was also examined descriptively, and a matched survival comparison was performed after excluding MM-first lung cancer cases and their corresponding controls using a log-rank test stratified by matched set. Cox regression was not performed for these exploratory subgroup analyses because of the small subgroup sizes and limited number of events. Analyses were based on available data, with no imputation for missing values. All tests were two-sided, with P < 0.05 considered statistically significant. Statistical analyses were performed using R version 4.3.3 and GraphPad Prism version 10.1.2.

### 2.8 Ethics

This was a single-center retrospective medical record study that did not involve any intervention in patient care. All data were deidentified before analysis, and no patient names, national identification numbers, hospital identification numbers, contact details, or other personally identifiable information are presented in the manuscript or figures.

The study was approved by the Ethics Committee of the Affiliated Hospital of Shandong University of Traditional Chinese Medicine (approval No. 2026-155-01-KY). Because this was a retrospective study involving no more than minimal risk, the requirement for informed consent was waived by the ethics committee.

## 3 Results

### 3.1 Patient selection and construction of the matched cohort

During the study period, 461 unique patients with MM were identified after deduplication and constituted the overall screening cohort. Of these, 27 patients had an OPM diagnosed either before, concurrently with, or after MM, corresponding to a crude proportion of 5.9%. These OPMs comprised 25 solid tumors and 2 cases of diffuse large B-cell lymphoma (DLBCL). This value represents the crude proportion of patients with an OPM among unique patients with MM treated or followed at our center and should not be interpreted as a population-based incidence rate or a cumulative incidence of second primary malignancies.

**Figure 1:**
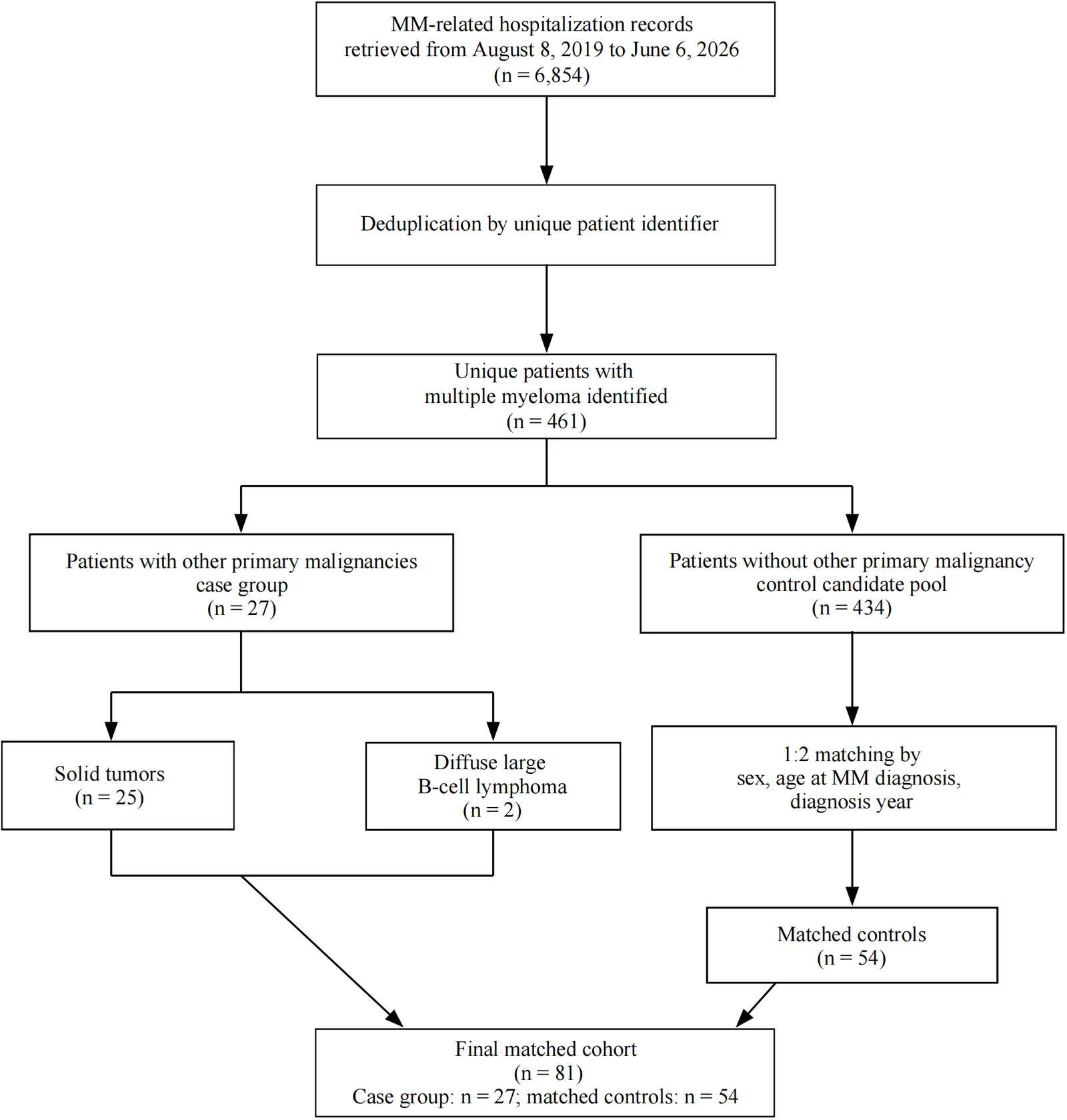
Patient selection and construction of the matched cohort. MM-related inpatient records from August 8, 2019, to June 6, 2026, were retrieved from the medical record system. After duplicate hospitalization records were removed and records were deduplicated using unique patient identifiers, 461 patients with MM were identified. Among them, 27 patients had an OPM and comprised the case group, including 25 patients with solid tumors and 2 with DLBCL. The remaining 434 patients with MM without an OPM constituted the pool of potential controls. After 1:2 matching by sex, age at MM diagnosis, and year of MM diagnosis, 54 matched controls were selected, resulting in a matched cohort of 27 cases and 54 controls for baseline comparisons and OS analysis. DLBCL, diffuse large B-cell lymphoma; MM, multiple myeloma; OPM, other primary malignancy; OS, overall survival.

Matched controls were subsequently selected from contemporaneous patients with MM without an OPM. After matching by sex, age at MM diagnosis, and year of MM diagnosis, the final matched cohort comprised 27 cases and 54 matched controls and was used for subsequent comparisons of baseline characteristics and survival outcomes. The patient selection process and construction of the matched cohort are shown in **Figure 1**.

### 3.2 Baseline characteristics of the matched cohort

The matched cohort included 81 patients with MM: 27 cases with an OPM and 54 matched controls. The case group included 16 men (59.3%) and 11 women (40.7%), whereas the control group included 32 men (59.3%) and 22 women (40.7%). The median age at MM diagnosis was 66 years (range, 56–78 years) in the case group and 67 years (range, 51–80 years) in the control group. The median year of MM diagnosis was 2022 in both groups. As expected, the groups were comparable with respect to the matching variables.

The distributions of MM-related clinical characteristics, including R-ISS stage, hemoglobin level, bone marrow plasma cell percentage, extramedullary disease at MM diagnosis, frontline anti-myeloma treatment, and receipt of autologous stem cell transplantation, are shown in **Table 1**. Frontline treatment was further summarized according to the use of proteasome inhibitor-, lenalidomide-, and anti-CD38 monoclonal antibody–containing regimens. Specific frontline anti-myeloma regimens are provided in **Supplementary Table 1**; VD (bortezomib plus dexamethasone) and VRD (bortezomib plus lenalidomide and dexamethasone) were the most frequently used regimens in both groups.

**Table 1:** Baseline characteristics and MM treatment in the matched cohort.

Variable	Cases (n = 27)	Matched controls (n = 54)	*P* value
Sex			Matching variable
Male	16 (59.3)	32 (59.3)	
Female	11 (40.7)	22 (40.7)	
Age at MM diagnosis, y	66 (56–78)	67 (51–80)	Matching variable
Year of MM diagnosis	2022 (2012–2025)	2022 (2012–2026)	Matching variable
R-ISS stage			0.253
I	1 (3.7)	7 (13.0)	
II	18 (66.7)	27 (50.0)	
III	8 (29.6)	20 (37.0)	
Hemoglobin, g/L	92 (67–118)	103 (21–147)	0.233
Bone marrow plasma cells, %	22.0 (11.5–60.0)	22.5 (8.0–93.0)	0.386
Extramedullary disease at MM diagnosis, yes, n (%)	3 (11.1)	4 (7.4)	0.681
Proteasome inhibitor–containing frontline regimen, n (%)	24 (88.9)	52 (96.3)	0.327
Lenalidomide-containing frontline regimen, n (%)	11 (40.7)	20 (37.0)	0.811
Anti-CD38 monoclonal antibody–containing frontline regimen, n (%)	2 (7.4)	2 (3.7)	0.597
ASCT, n (%)	6 (22.2)	10 (18.5)	0.770

Data are presented as n (%) unless otherwise specified. Continuous variables are presented as median (range). Sex, age at MM diagnosis, and year of MM diagnosis were matching variables and were not subjected to between-group statistical testing. Frontline treatment categories were not mutually exclusive because a single regimen could contain more than one drug class. Continuous variables were compared using the Mann–Whitney U test. R-ISS stage was compared using the Pearson χ² test, whereas binary categorical variables were compared using Fisher’s exact test. P values for nonmatching variables are provided for descriptive comparison only. ASCT, autologous stem cell transplantation; MM, multiple myeloma; R-ISS, Revised International Staging System.

### 3.3 Spectrum and clinical characteristics of other primary malignancies

**Figure 2:**
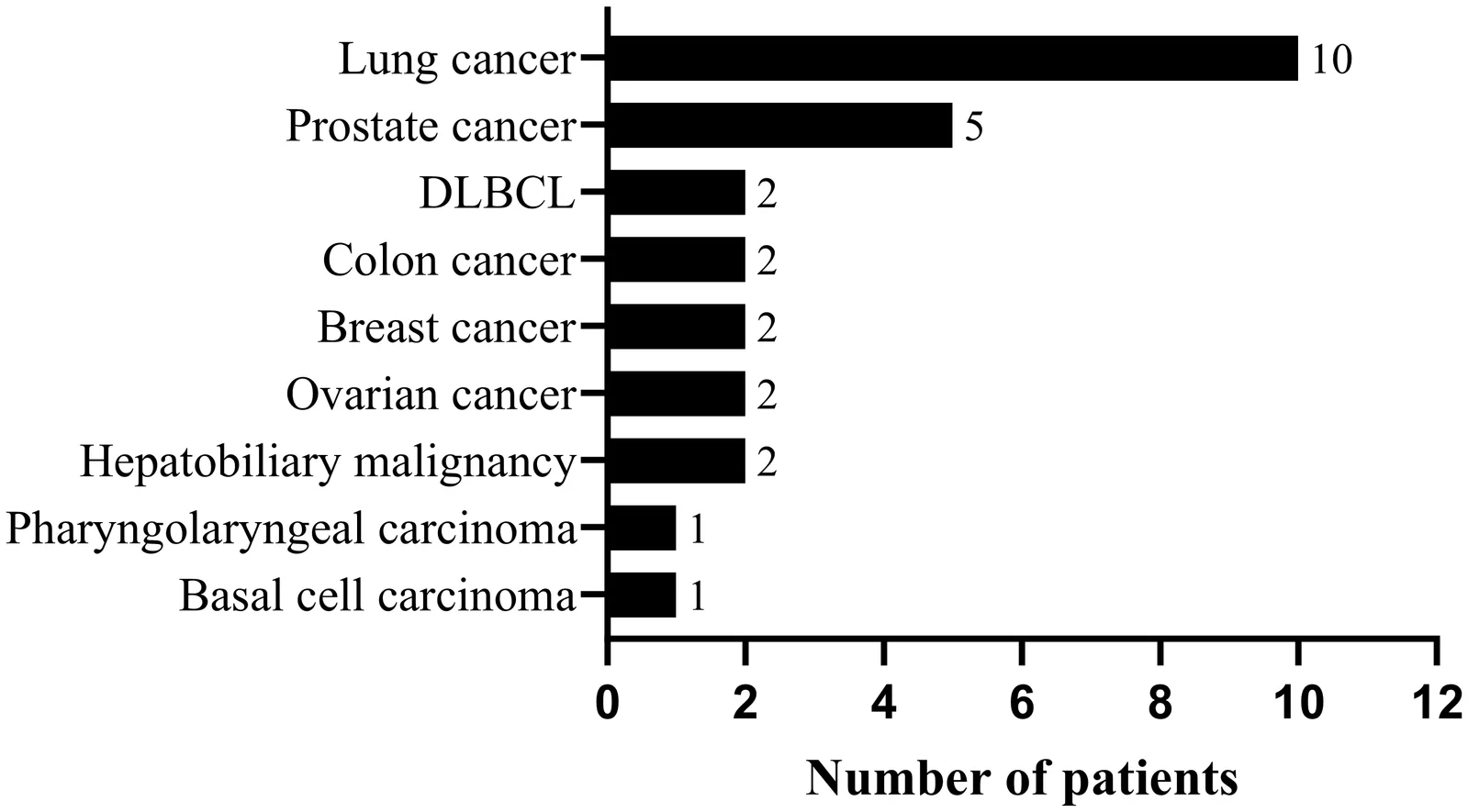
Distribution of other primary malignancies among 27 patients with multiple myeloma. Lung cancer was the most common OPM, followed by prostate cancer. DLBCL, diffuse large B-cell lymphoma; MM, multiple myeloma; OPM, other primary malignancy.

Among the 27 patients with an OPM, 25 (92.6%) had a solid tumor and 2 (7.4%) had diffuse large B-cell lymphoma (DLBCL). Lung cancer was the most common OPM, occurring in 10 patients (37.0%), including 6 with adenocarcinoma, 3 with squamous cell carcinoma, and 1 with small-cell lung cancer. Prostate cancer was the second most common OPM, occurring in 5 patients (18.5%). The distribution of the remaining OPM types is shown in **Figure 2**.

Among the 22 patients in whom the OPM had been diagnosed by the time of MM diagnosis, 14 had active disease, whereas 8 had a previously treated or controlled malignancy with no documented active disease at MM diagnosis. The remaining 5 patients developed an OPM after MM diagnosis. Among the 25 patients with solid tumors, 7 (28.0%) had documented distant metastasis at OPM diagnosis. These clinical characteristics are summarized in **Table 2**.

**Table 2:** Clinical characteristics of other primary malignancies in the case cohort.

Item	Category	No. (%)
Tumor category	Solid tumor	25 (92.6)
	Diffuse large B-cell lymphoma	2 (7.4)
OPM status at MM diagnosis	Active OPM	14 (51.9)
	Previously treated or controlled OPM	8 (29.6)
	Not yet diagnosed (MM-first)	5 (18.5)
Distant metastasis at diagnosis	Yes	7 (25.9)
	No	18 (66.7)
	Not applicable (DLBCL)	2 (7.4)

Data are presented as n (%), with all 27 patients with MM and an OPM used as the denominator. Distant metastasis was assessed for solid tumors only; the two patients with diffuse large B-cell lymphoma were classified as not applicable. DLBCL, diffuse large B-cell lymphoma; MM, multiple myeloma; OPM, other primary malignancy.

### 3.4 Temporal relationship between MM and other primary malignancies

Using the prespecified 2-month cutoff, 10 of the 27 patients (37.0%) were classified as OPM-first, 12 (44.4%) as synchronous, and 5 (18.5%) as MM-first. The median interval between OPM and MM diagnoses was 0 months (range, −235 to 66 months).

**Figure 3:**
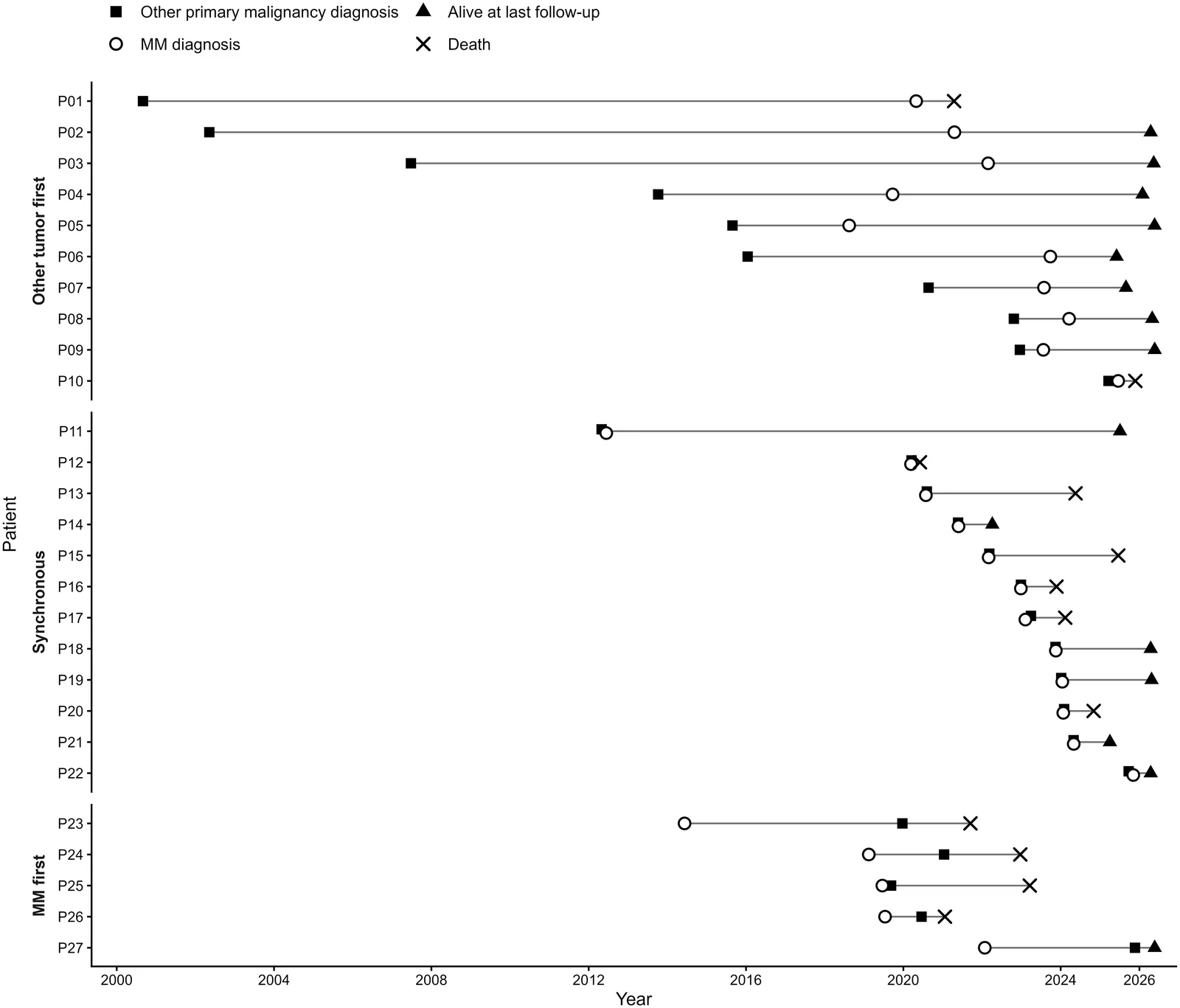
Patient-level timeline of MM and OPM diagnoses and follow-up. Each horizontal line represents one patient and extends from the earlier of the two malignancy diagnoses to death or the last follow-up. Filled squares indicate OPM diagnosis, open circles indicate MM diagnosis, filled triangles indicate that the patient was alive at the last follow-up, and crosses indicate death. For patients with closely spaced MM and OPM diagnoses, the two diagnostic markers were slightly offset vertically to improve visibility, without altering their positions on the time axis. Patients are grouped according to the temporal relationship between MM and OPM. MM, multiple myeloma; OPM, other primary malignancy.

The OPM-first group showed considerable variation in diagnostic interval, with several OPMs diagnosed more than 10 years before MM. Because a long diagnostic interval did not necessarily indicate active malignancy at the time of MM diagnosis, OPM status at MM diagnosis was assessed separately and is summarized in **Table 2**. The diagnosis dates of both malignancies and subsequent follow-up for all 27 patients are shown in **Figure 3**.

### 3.5 Treatment patterns and interactions between MM and OPM management

Among the 27 patients with an OPM, OPM treatment was initiated first in 13 patients (48.1%), MM treatment was initiated first in 11 (40.7%), and the two malignancies were treated concurrently in 3 (11.1%). Eleven patients (40.7%) underwent surgery for the OPM, and 24 (88.9%) received systemic antineoplastic therapy.

The presence of an OPM affected MM management in 8 patients (29.6%). The main changes involved treatment timing or sequencing in 5 patients and modification of the MM regimen or treatment intensity in 3 patients. In 4 patients, the ASCT plan was modified or ASCT was not undertaken in the context of the OPM and its treatment. Conversely, MM or anti-myeloma treatment affected OPM management in 5 patients (18.5%), all of whom required adjustment of the timing or sequencing of OPM-directed treatment.

Treatment was modified because of documented or anticipated overlapping toxicities in 8 patients (29.6%). Bone marrow suppression was the most frequently documented concern, often accompanied by renal toxicity, peripheral neuropathy, infection risk, or anemia. Treatment patterns and interactions between MM and OPM management are summarized in **Table 3**.

**Table 3:** Treatment patterns and interactions between MM and OPM management.

Item	Category	No. (%)
Treatment sequencing	OPM treated first	13 (48.1)
	MM treated first	11 (40.7)
	Concurrent treatment	3 (11.1)
Surgery for the OPM	Yes	11 (40.7)
	No	16 (59.3)
Systemic antineoplastic therapy for the OPM	Yes	24 (88.9)
	No	3 (11.1)
OPM affected MM management	Yes	8 (29.6)
	No	18 (66.7)
	Unknown	1 (3.7)
MM affected OPM management	Yes	5 (18.5)
	No	19 (70.4)
	Unknown	3 (11.1)
Treatment modification related to overlapping toxicities	Yes	8 (29.6)
	No	19 (70.4)

Data are presented as n (%). “Unknown” indicates that the available records were insufficient to determine whether one malignancy affected the management of the other. Treatment modifications related to overlapping toxicities included changes made because of documented adverse events or anticipated toxicity overlap between the two treatment approaches. MM, multiple myeloma; OPM, other primary malignancy.

### 3.6 Overall survival in the matched cohort

During follow-up, 12 deaths occurred among the 27 cases and 9 among the 54 matched controls. Median OS from the date of MM diagnosis was 46.2 months in the case group and was not reached in the matched control group. The 1-, 3-, and 5-year OS rates were 80.5%, 72.0%, and 45.8%, respectively, in the case group and 96.0%, 88.6%, and 71.0%, respectively, in the matched control group.

**Figure 4:**
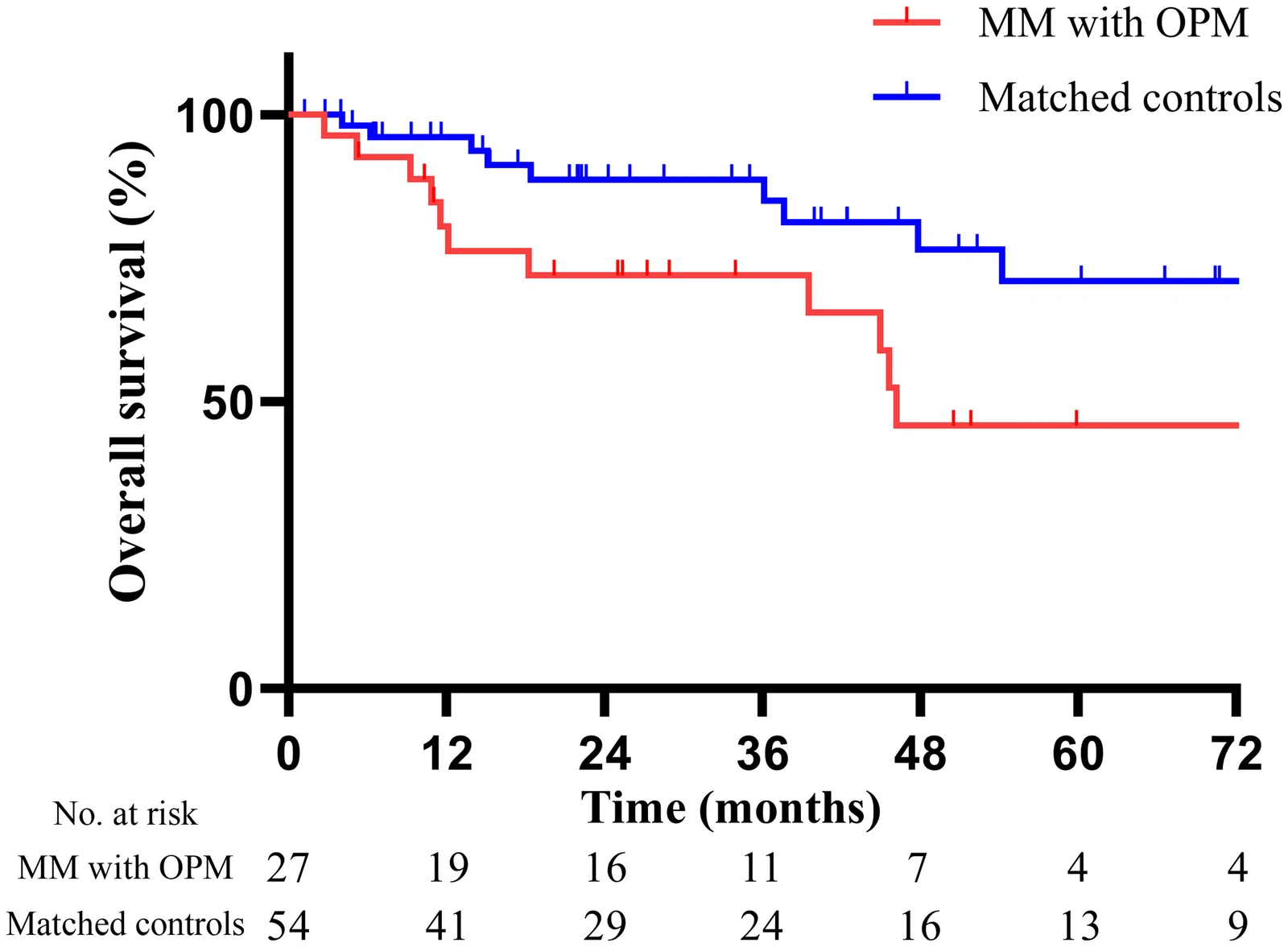
Overall survival in cases and matched controls. OS was measured from the date of MM diagnosis. Cases were patients with MM and an OPM, whereas controls were patients with MM without an OPM who were matched by sex, age at MM diagnosis, and year of MM diagnosis. Median OS was 46.2 months in the case group and was not reached in the matched control group. Between-group differences were assessed using a log-rank test stratified by matched set, which showed inferior OS in the case group (*P* = 0.007). MM, multiple myeloma; OPM, other primary malignancy; OS, overall survival.

Cases had inferior OS compared with matched controls. The matched-set-stratified log-rank test showed a between-group difference in OS (P = 0.007), while the unstratified log-rank test yielded P = 0.018. In the Cox proportional hazards model stratified by matched set, cases had a higher hazard of death than matched controls (hazard ratio, 3.92; 95% CI, 1.35–11.37; P = 0.012). The OS curves for the matched cohort are shown in **Figure 4**.

### 3.7 Sensitivity and exploratory analyses

To reduce the potential influence of MM-first cases on the analysis of OS measured from MM diagnosis, 5 MM-first cases and their corresponding 10 matched controls were excluded. The resulting sensitivity-analysis cohort comprised 22 cases and 44 matched controls. Eight deaths occurred in the case group and 6 in the control group. Median OS was not reached in either group. The 1-, 3-, and 5-year OS rates were 75.7%, 70.3%, and 50.2%, respectively, in the case group and 95.1%, 89.0%, and 79.1%, respectively, in the control group.

The unstratified log-rank test showed inferior OS in the case group (*P* = 0.042). The log-rank test stratified by matched set also demonstrated a statistically significant between-group difference (*P* = 0.035). In the Cox proportional hazards model stratified by matched set, cases had a higher hazard of death than matched controls (hazard ratio, 3.42; 95% CI, 1.02–11.48; *P* = 0.047). These findings indicate that the association between an OPM and inferior OS persisted after MM-first cases were excluded. Survival outcomes from the primary and sensitivity analyses are summarized in **Table 4**.

**Table 4:** Overall survival outcomes in the primary and sensitivity analyses.

Analysis	Cases, n (deaths)	Matched controls, n (deaths)	Median OS, months (cases vs matched controls)	Stratified log-rank *P*	Stratified Cox HR (95% CI), *P*
Primary analysis	27 (12)	54 (9)	46.2 vs NR	0.007	3.92 (1.35–11.37), 0.012
Sensitivity analysis excluding MM-first cases	22 (8)	44 (6)	NR vs NR	0.035	3.42 (1.02–11.48), 0.047

OS was measured from the date of MM diagnosis. Median OS values are shown for cases versus matched controls. HRs compare cases with matched controls. The sensitivity analysis excluded 5 MM-first cases and their 10 corresponding matched controls. The reported log-rank P values and Cox proportional hazards models were stratified by matched set. CI, confidence interval; HR, hazard ratio; MM, multiple myeloma; NR, not reached; OPM, other primary malignancy; OS, overall survival.

An exploratory analysis was also performed according to OPM status at the time of MM diagnosis. Among the 22 patients whose OPM had been diagnosed by the time of MM diagnosis, 14 had active OPM and 8 had previously treated or controlled OPM. Seven deaths occurred in the active OPM group and 1 in the previously treated or controlled OPM group. Median OS was 39.6 months in the active OPM group and was not reached in the previously treated or controlled OPM group. Patients with active OPM had shorter OS than those with previously treated or controlled OPM (log-rank P = 0.036). Given the small subgroup sizes and limited number of events, Cox regression was not performed.

### 3.8 Exploratory analysis of the lung cancer subgroup

Among the 10 patients with lung cancer, 2 were classified as OPM-first, 6 as synchronous, and 2 as MM-first. Five patients had distant metastasis at lung cancer diagnosis. OPM-directed treatment was initiated first in 2 patients, MM treatment was initiated first in 5, and treatment for the two malignancies was administered concurrently in 3. During follow-up, 5 patients died, and median OS from MM diagnosis was 45.7 months.

To reduce the potential influence of lung cancers diagnosed after MM, the 2 MM-first lung cancer cases and their 4 corresponding matched controls were excluded from an exploratory matched analysis. The resulting cohort included 8 lung cancer cases and 16 matched controls, with 4 and 1 deaths, respectively. Median OS was 45.7 months in the lung cancer group and was not reached in the matched control group. The log-rank test stratified by matched set showed shorter OS in the lung cancer group (P = 0.042). Because of the small subgroup size and limited number of events, Cox regression was not performed.

## 4 Discussion

In this single-center cohort of 461 patients with MM, we identified 27 patients with an OPM and subsequently constructed a 1:2 matched control cohort to evaluate survival outcomes. Most OPMs were solid tumors, with lung cancer and prostate cancer being the most common, and the majority were diagnosed before or concurrently with MM. Patients in the case group had inferior OS compared with matched controls, and this difference persisted after MM-first cases and their corresponding controls were excluded. In an exploratory analysis restricted to patients whose OPM had been diagnosed by the time of MM diagnosis, those with active OPM had shorter OS than those with previously treated or controlled OPM. These findings illustrate the heterogeneity of OPMs in patients with MM with respect to diagnostic timing, disease activity, and survival outcomes.

Previous studies of second primary malignancies in patients with MM have largely focused on malignancies arising after treatment, with particular attention to treatment exposure, host-related factors, disease-associated immune dysfunction, and prolonged follow-up (5–7). In our cohort, however, only 5 of 27 patients (18.5%) were classified as MM-first, whereas the remaining OPMs were diagnosed before or concurrently with MM. These malignancies therefore cannot be uniformly regarded as treatment-emergent second primary malignancies. We used the term “other primary malignancy” to describe independent primary malignancies diagnosed before, concurrently with, or after MM without implying a causal relationship with anti-myeloma treatment. Previous single-center and population-based studies have likewise reported considerable heterogeneity in both tumor type and the temporal sequence of MM and other malignancies (10–13).

The substantial proportion of synchronous diagnoses was another notable feature of this cohort. Temporal proximity between MM and an OPM does not necessarily indicate a biological relationship between the two malignancies and may instead reflect their detection during the same diagnostic evaluation. Because manifestations of MM may overlap with those of metastatic solid tumors or other hematologic malignancies, distinguishing an independent primary malignancy from MM-related lesions or metastatic disease is clinically important. Histopathologic confirmation, together with assessment of lesion distribution, imaging findings, and the clinical course, remains essential when an additional primary malignancy is suspected (8, 14).

Treatment interactions between MM and OPM were frequent and bidirectional in this cohort. The presence of an OPM affected MM management in 8 patients, mainly through changes in treatment timing or sequencing and modifications to the regimen or treatment intensity; in 4 patients, the ASCT plan was modified or ASCT was not undertaken in the context of the OPM and its treatment. Conversely, MM or anti-myeloma treatment affected OPM management in 5 patients, all of whom required adjustment of the timing or sequencing of OPM-directed treatment. Treatment was also modified in 8 patients because of documented or anticipated overlapping toxicities, most commonly bone marrow suppression, with renal toxicity, peripheral neuropathy, infection risk, and anemia also contributing to treatment decisions. Although no statistically significant between-group differences were observed in the recorded frontline MM treatment classes or ASCT receipt, these patient-level treatment interactions indicate that similar overall treatment distributions do not preclude clinically meaningful modifications in individual patients. Treatment priority should therefore be individualized according to the activity and urgency of both malignancies, anticipated treatment-related toxicities, and the patient’s overall condition (15). MM-related disease burden and clinically important complications, including renal impairment and bone disease, should also be considered when determining treatment urgency (16–18). Multidisciplinary coordination is particularly important when treatment for both malignancies is required within the same period.

The principal survival finding of this study was the inferior OS observed in the case group compared with matched controls. This difference remained after MM-first cases and their corresponding controls were excluded. However, the pooled hazard ratio should be interpreted as an association observed across this heterogeneous cohort rather than as a uniform effect attributable to any specific type of OPM. The case group included malignancies with markedly different biological behavior, stage, treatment, and prognosis, and the contributions of individual tumor types to the overall survival difference could not be reliably separated. In an exploratory analysis of lung cancer, the most common OPM in this cohort, a similar pattern of shorter OS was observed relative to matched controls after MM-first lung cancer cases and their corresponding controls were excluded. However, the small subgroup size and limited number of events preclude a definitive tumor-specific interpretation of this finding. The exploratory analysis according to OPM status at MM diagnosis further showed shorter OS in patients with active OPM than in those with previously treated or controlled OPM, suggesting that disease activity at the time of MM diagnosis may also contribute to the observed heterogeneity. Together, these exploratory findings suggest that both tumor type and disease activity may contribute to heterogeneity in survival, although their individual contributions to the pooled survival association cannot be quantified in this small cohort. These findings support an association between OPMs and inferior survival in this cohort but do not establish OPM status as an independent prognostic factor or imply causality. Previous studies have also reported adverse outcomes among patients with MM who develop second primary malignancies (19), while population-based studies indicate that non-MM causes of death, including other cancers, contribute substantially to overall mortality in MM (20, 21).

This study has several limitations. First, it was a single-center retrospective study with only 27 cases encompassing a wide range of OPM types with markedly different biological behavior, treatment, and prognosis. The pooled survival estimate therefore reflects an association across a heterogeneous cohort and should not be interpreted as a tumor-specific effect. Exploratory analyses according to OPM status at MM diagnosis and within the lung cancer subgroup were also constrained by small subgroup sizes and limited numbers of events. Second, although the matched design accounted for sex, age at MM diagnosis, and year of MM diagnosis, residual confounding by MM-related prognostic factors, comorbidities, disease burden, treatment selection, and other unmeasured factors may remain. Extramedullary disease, frontline treatment classes, and ASCT were evaluated descriptively, but the limited number of deaths precluded reliable multivariable adjustment for these factors. Third, the small number of patients remaining at risk during later follow-up limits the precision of long-term OS estimates. Finally, B-cell receptor sequencing or other clonality data were not available for the two patients with DLBCL, precluding assessment of a potential clonal relationship between MM and DLBCL. Larger multicenter studies with more homogeneous tumor subgroups, more events, and comprehensive molecular data are needed to validate these findings and better define tumor-specific outcomes.

## 5 Conclusion

Lung and prostate cancers were the most common OPMs in this cohort, highlighting the need for heightened clinical attention to these malignancies in patients with MM. The presence of an OPM was associated with inferior OS; however, this finding should be interpreted cautiously because of the small sample size and marked tumor heterogeneity. When both malignancies require active treatment, multidisciplinary assessment is important to determine treatment priority according to disease activity, treatment urgency, and the patient’s overall condition.

## Data Availability

The datasets presented in this article are not readily available because they contain potentially sensitive patient-level clinical information and are subject to institutional and patient-privacy restrictions. Requests to access the datasets should be directed to the corresponding author. Deidentified data may be made available upon reasonable request, subject to applicable institutional and ethical requirements.
