# Supplementary material for: Other primary malignancies are associated with inferior survival in multiple myeloma: a single-center matched retrospective cohort study
Source: Front Oncol. 2026 Sep 11;16:1943171. doi: 10.3389/fonc.2026.1943171 (PMC13612320; doi:10.3389/fonc.2026.1943171)
Supplement: Supplementary file 1 [file Table1.docx]

**Supplementary Table S1. Frontline Anti-myeloma Regimens in the Matched Cohort**

| **Frontline regimen** | **Cases (n = 27), n (%)** | **Matched controls (n = 54), n (%)** |
| --- | --- | --- |
| VD | 12 (44.4) | 23 (42.6) |
| VRD | 8 (29.6) | 18 (33.3) |
| VCD | 2 (7.4) | 10 (18.5) |
| DRD | 2 (7.4) | 2 (3.7) |
| PAD | 2 (7.4) | 1 (1.9) |
| RD | 1 (3.7) | 0 (0) |

Data are presented as n (%). Frontline regimen categories are mutually exclusive. Percentages may not total 100% because of rounding. VD, bortezomib plus dexamethasone; VRD, bortezomib plus lenalidomide and dexamethasone; VCD, bortezomib plus cyclophosphamide and dexamethasone; DRD, daratumumab plus lenalidomide and dexamethasone; PAD, bortezomib plus doxorubicin and dexamethasone; RD, lenalidomide plus dexamethasone.
